# Periprosthetic Fractures after Total Knee Arthroplasty: the Influence of Pre-Operative Mechanical Factors versus Intraoperative Factors

**DOI:** 10.5704/MOJ.1907.005

**Published:** 2019-07

**Authors:** S Zainul-Abidin, BTJ Lim, HR Bin-Abd-Razak, C Gatot, JC Allen, JSB Koh, TS Howe

**Affiliations:** Department of Orthopaedic Surgery, Singapore General Hospital, Singapore; * Centre for Quantitative Medicine, DukeNUS Medical School, Singapore

**Keywords:** periprosthetic fracture, total knee arthroplasty, notching, valgus knee, range of motion

## Abstract

**Introduction:** Periprosthetic fractures are a devastating complication following total knee arthroplasty. Little is known about the effect of mechanical factors on the incidence of periprosthetic fractures. The aim of this study was to examine the correlation between pre-operative mechanical factors, like side of surgery, coronal alignment and pre-operative range of motion and intra-operative factors, and the incidence of a periprosthetic fracture, following primary total knee arthroplasty (TKA).

**Materials and Methods:** Forty-two patients with periprosthetic fractures (PPF) after primary TKA were identified from our hospital arthroplasty registry. These patients were matched two-to-one for gender and age at primary knee arthroplasty to 84 patients without PPF. The incidence of periprosthetic fracture with regards to laterality, coronal alignment and pre-operative range of motion was analysed. Intra-operative factors like implant type, patellar resurfacing and notching were also analysed using logistic regression.

**Results:** Coronal alignment, pre-operative range of motion and patella resurfacing were not significant predictors of periprosthetic fractures. Anterior femoral notching was found to be significantly higher in the fracture group with an odds ratio of 17. Left sided surgery was also significantly higher in the periprosthetic fracture group.

**Conclusion:** Periprosthetic fractures are 17 times more likely to occur in a knee with anterior femoral notching. Preoperative factors like coronal alignment and poor preoperative range of motion do not seem to increase the risk of periprosthetic fractures after TKA.

## Introduction

Total Knee Arthroplasty (TKA) is widely regarded as a safe, effective and rewarding procedure, with patients consistently reporting excellent satisfaction rates ranging from 78% to 94%^1^. Periprosthetic fractures (PPF) however, are a devastating complication for both the patients and surgeons, potentially rendering the patient immobile, drastically reducing their function and also frequently resulting in multiple surgical procedures, increased duration of hospital stay as well as additional hospital expense. The minority of TKA patients who suffer from periprosthetic fractures unfortunately eventually end up having poorer functional outcome scores and satisfaction rates^2^.

Preoperative risk factors that have been reported in the literature thus far include, previous revision TKA, prolonged steroid use, rheumatoid arthritis, advanced age, female gender, neurologic disorders and, controversially, the presence of anterior femoral notching^3^-^5^. However, the effect of pre-operative factors on periprosthetic fractures is less well documented. Limited analysis of the outcomes of patients with poor pre-operative range of motion (ROM) undergoing knee arthroplasty found that there was a much higher complication rate of 41% and a high revision rate of 18.5%, including revisions secondary to PPF^6^. Other potential mechanical factors such as coronal leg alignment (pre-operative varus or valgus deformity) and leg side dominance on the incidence of periprosthetic fractures have not been studied in depth though some studies suggested that varus malalignment was associated with periprosthetic fractures^7^.

Our null hypothesis is that mechanical factors such as coronal alignment, leg dominance and pre-operative range of motion are similar between patients with and without periprosthetic fractures. Our study aimed to compare preoperative mechanical factors (including coronal leg alignment and pre-operative range of motion) between patients sustaining periprosthetic fractures after TKR against a gender and age-matched control group with no periprosthetic fracture. These factors were also analysed against the potential effects of intra-operative technical confounders such as anterior femoral notching and implant constraint.

## Materials and Methods

The ‘Case’ group (or ‘fracture’ group) of 42 patients with periprosthetic fracture following primary TKA was identified from prospectively collected data in the hospital arthroplasty registry between 2000 and 2015. All patients underwent primary total knee arthroplasty by a fellowship-trained orthopaedic specialist surgeon and were placed on a standardised postoperative arthroplasty clinical pathway and physiotherapy regime in our institution. All patients with a lower limb deformity not related to the primary surgery and/or neurological deficit impairing the function of the operated limb were excluded from the study.

These 42 patients were matched 2:1 for gender and age at primary knee arthroplasty to 84 patients without periprosthetic fracture after TKA (‘Control’ or ‘non fracture’ group). Pre-operative patient demographics (age, gender, BMI), pre-operative range of motion, coronal alignment (varus or valgus deformity) and radiographs were analysed for all patients.

To determine coronal leg alignment and range of motion, all patients were assessed pre-operatively by a specially trained group of physiotherapists. Knee alignment was measured using photogrammetry: in this technique, bony processes were identified by manual palpation. The anterior superior iliac spine (ASIS), centre of patella and talar head were identified in accordance with the recommendations of Gross *et al*^8^ . Spherical markers were attached at these anatomical points. The patients were then photographed. Frontal views were analysed by our AutoCAD 2000 software to measure the angles between the thigh and lower leg segments. In the frontal views, varus / valgus angles of the knee were drawn using two straight lines, one joining the ASIS to the centre of the patella, and the other joining the centre of the patella to the talar head^9^. Range of motion of the knee was measured using a goniometer, and measuring the angles at the knee during maximal flexion and extension.

All patients were planned for a primary total knee replacement via a parapatellar approach using either cruciate-retaining or posterior-stabilized implants, with cementing of the prostheses in all patients. Not all patellae were resurfaced and this was determined by surgeon’s preference as well as the size of the patella intraoperatively. All patients had mechanical and/or chemo - prophylaxis to prevent venous thromboembolism post-operatively.

Postoperatively, patients were assessed by a physiotherapist daily. All patients were allowed to ambulate with a walking aid by the second postoperative day and were discharged home once they were able to actively flex the knee to 90 degrees, to perform unassisted straight-leg raising, to ambulate independently and to climb stairs (if they were required to do so at their homes). Patients who required additional rehabilitation were discharged to a community rehabilitation hospital. All patients underwent outpatient physiotherapy until their progress was deemed satisfactory for discharge.

Patients with periprosthetic fractures were contacted by telephone retrospectively to enquire on their leg dominance (“Which leg do you use first to climb up stairs?”). Leg dominance data was available for 33 out of the 42 patients. With regards to anterior femoral notching, radiographs of all the patients were reviewed by three of the authors (SZA, LBTJ and CG). The presence of anterior femoral notching in post-operative radiographs was ascertained and graded according to the Tayside Classification: Grade I (violation of the outer table of the anterior femoral cortex), Grade II (violation of the outer and inner table to the centre of the femoral cortex), Grade III (violation of up to 25% of the medullary canal) or Grade IV (violation of up to 50% of the medullary canal)^10^.

Statistical analysis was carried out using paired t-test for continuous variables between the study and control group. The incidence of periprosthetic fractures with regard to fixed flexion deformity (FFD), flexion limitation, varus malalignment or valgus malalignment was analysed using a chi-squared test. Implant type, the presence of femoral notching, patellar resurfacing and proportion of RA versus OA were also analysed using the Chi-squared test.

Variables of patient demographics, ROM, side of surgery and coronal alignment were entered into a univariate logistic regression model. Variables with p-value < 0.2 were then entered into a multivariate logistic regression model in a stepwise manner. A variable was considered to be a significant predictor if its odds ratio was significant at p < 0.05 after multiple regression analysis. All statistical analyses were performed using SPSS version 21 (SPSS, Inc., Chicago, Illinois) in consultation with a biostatistician (AJC).

Power analysis was performed to ensure that the study sample size was adequate. A post hoc power analysis using 126 patients as the sample size (total number of patients in the study), and an alpha value of 0.05 showed a power of 100%. This study was designed as a retrospective case-control study. This study was exempted from IRB ethical approval as no patients were directly involved in the study and all data was anonymised.

## Results

There were 42 patients with periprosthetic fractures following primary TKA (37 female and five male). These patients were matched 2:1 for age at TKA and gender to 84 patients (74 female and 10 male). Patient demographics are shown in Table I.

**Table I:** Patient demographics and clinical details

Patient Demographics and Clinical Details	Cases (PPF)	Controls (TKA with no PPF)
Number of patients	42	84
Male	5	10
Female	37	74
Age at surgery	68.9 ± 8.2	68.9 ± 8.2
Body Mass Index	28.4 ± 4.8	27.5 ± 3.9
Side of surgery : Left	26	36
Side of Surgery : Right	16	48
Alignment : Varus	25	62
Alignment : Valgus	16	22
Alignment : Neutral	1	0
Pre-operative extension (mean)	9.3 ± 13.4	8.1 ± 8.7
Pre-operative flexion (mean)	112.4 ± 24.2	116.7 ± 17.6
Notching	10	3
CR implants	27	57
PS implants	14	25
Rheumatoid Arthritis	2	1
Osteoarthritis	39	83

There was no difference in mean age at primary TKA for both groups (68.9 ± 8.2 years). BMI for both groups of patients was also not significantly different (28.4 ± 4.8 for PPF group vs 27.5 ± 3.9 for control group, p=0.425). Of the 42 PPFs, 40 were femoral fractures (supracondylar region of distal femur) whilst two were tibial fractures. Mean time period from primary TKA to occurrence of periprosthetic fracture was 22.8 ± 30.5 months, with 29 early ( less than two years after surgery) and 12 late fractures (more than two years after surgery).

Low velocity injury was the cause for all the peri-prosthetic fractures: 21 patients had fallen on slippery floor, 12 patients had tripped on uneven grounds/stairs and nine patients had twisted their knee while getting up from a lying/sitting position. Four patients also had a history of cerebrovascular accident, which could have predisposed to the falls.

The mean age of the 42 patients at the time of fracture was 71 years. In the periprosthetic fracture group, 39 patients were diagnosed with osteoarthritis, two with rheumatoid arthritis and one with avascular necrosis. In the non-fracture group, 83 patients had osteoarthritis whilst only one had rheumatoid arthritis. There was no statistically significant difference between the proportion of osteoarthritis and rheumatoid arthritis patients in both groups (Chi-square statistic 1.60, p-value = 0.206). From radiological assessments, there were 10 patients in the PPF group who had anterior femur notching after their primary TKA. Based on Tayside's classification, there were four patients with grade I notching, three patients with grade II notching and three patients with Grade III notching. Only three of the ten patients had fracture through their notching (two patients had Grade I notching and one had Grade III notching), which was not significant (p=0.073). Only three patients in the non-fracture group were found to have anterior femoral notching. Univariate logistic regression showed this to be significantly different between the fracture and non-fracture group (p=0.0019, odds ratio 10.66). On multiple logistic regression, notching remained highly significant at p=0.0008, with an odds ratio of 17.43.

For the fracture group, the types of implants used were: 27 cruciate- retaining, 14 posterior-stabilised and one constrained knee (due to femoral notching). For the non-fracture group, there were 25 cruciate retaining implants, 57 posterior-stabilised implants and two constrained knee implants used (also due to femoral notching). There were significantly more posterior-stabilised implants in the non-fracture group (Chi-square statistic 14.01, p = 0.000182).

Of the 42 knees in the fracture group, 17 had the patella resurfaced whilst 25 of the patellae were not. Of the 84 patellae in the non-fracture group, 29 were resurfaced whilst 55 of the patella were not resurfaced. This was not found to be significantly different between the two groups (Chi-square statistic 0.3675, p = 0.544).

Patients in the Peri-Prosthetic Fracture (PPF) group were found to have a mean fixed flexion deformity (FFD) of 9.3◦ compared to 8.1◦ in the control group. Univariate analysis revealed that pre-operative FFD was not significantly different between the PPF and control group, with p-value = 0.240. The mean pre-operative flexion in patients with PPF was 112.4◦ compared to 116.7◦ for the control group. This was also found to be non-significant on univariate analysis, with p-value of 0.503.

The side of surgery was found to be a significant factor in predicting periprosthetic fractures, with a p-value of 0.085 on univariate logistic regression. When entered into a multivariate logistic regression, side of surgery remained a significant factor predicting PPF (p=0.0169, odds ratio 2.88).

Twenty-six of 42 (61.9%) patients in the PPF group had surgery on the left knee, whilst only 36 of 84 (42.9%) non-fracture patients had their TKA done on the left knee. We attempted to obtain data for leg dominance, via our retrospective data collection (phone interviews), and periprosthetic fractures were found to have occurred in the non-dominant leg in 17 of 33 contactable patients (52%). Five patients were deceased and four patients were uncontactable.

In terms of coronal alignment, 16 of 42 (38%) patients in the PPF group had a valgus alignment pre-operatively, 25 (59.5%) had varus alignment and 1 (2.3%) patient was neutral. For the control group, 62 out of 82 patients (73.8%) had varus alignment and 22 (26.9%) had valgus alignment. Pre-operative coronal alignment (varus, valgus or neutral) trended towards significance with univariate analysis of coronal alignment revealing a p-value of 0.189 for valgus knees, however it was found to be non-significant when entered into a multivariate logistic regression analysis.

A subgroup analysis of early and late periprosthetic fractures was also done: Pre-op BMI, diagnosis of OA or RA, pre-op alignment and pre-op range of motion were not found to be significant predictors of late or early periprosthetic fractures. However, the side of surgery was more significant in the early PPF group (p=0.0407 for the left side). Results of the statistical analysis and subgroup analyses are presented in Tables II and III.

**Table II:** Summary of statistical analysis

	Cases	Controls
FFD <10	25	48
FFD >10	17	34
Chi-Square statistic: 0.0112, p=0.916		
Flexion <90	6	7
Flexion > 90	36	77
Chi-Square statistic: 0.1772 , p=0.3004		
Posterior-Stabilised Implants	14	57
Cruciate-Retaining Implants	27	25
Chi-Square Statistic: 14.01, P=0.000182		
Osteoarthritis	39	83
Rheumatoid Arthritis	2	1
Chi-Square statistic: 1.60, p=0.206		
Patella resurfaced	17	25
Patella not resurfaced	24	59
Chi-Square statistic: 0.3675, p=0.544		
UNIVARIATE LOGISTIC REGRESSION	ODDS RATIO	P VALUE
Valgus Alignment	3.35	0.186
Left sided TKA	2.98	0.085
Pre-operative Extension	0.45	0.503
Pre-operative flexion	1.38	0.240
Notching	10.66	0.0019
MULTIVARIATE LOGISTIC REGRESSION	ODDS RATIO	P VALUE
Left Sided TKA	2.88	0.0169
Notching	17.43	0.0008

**Table III:** Subgroup analysis between early and late PPF

Univariate Logistic Regression	P Value
Pre-op Alignment (Valgus vs Varus)	0.305
Pre-op BMI	0.622
Notching	0.433
Poor pre-op flexion	0.310
Poor Pre-op Extension (FFD)	0.874
Side	0.0407

## Discussion

The rates of TKA are increasing worldwide and correspondingly, rates of associated complications like periprosthetic fractures as well^11^. Studies have reported the incidence of periprosthetic fractures to range between 0.3 and 5.5 % after primary TKA and even higher after revision TKA^12^-^13^.

With the added morbidity, mortality and expense that are incurred associated with periprosthetic fractures, numerous studies have been done to delineate the risk factors that predispose to this dreaded complication.

Our study found that periprosthetic fractures after TKA tended to occur in the supracondylar region of the femur and were mainly early complications occurring within two years of index surgery. Previous studies have shown that many knee replacements fail early: 35.3% of revisions occur at less than two years, and up to 60% in the first five years, with the leading cause of failure being attributed to aseptic loosening, and also citing periprosthetic fractures as a major cause of revisions^14^.

Our results showed that pre-operative mechanical factors such as coronal alignment and pre-operative range of motion were not found to significantly influence periprosthetic fractures. Valgus knees have been reported to have a high rate of patella stress fracture and osteonecrosis as compared to non-valgus TKA (1-12%)^9^ but our statistical analysis revealed that pre-operative valgus malalignment was not a significant risk factor for periprosthetic fracture. This finding is reassuring for arthroplasty surgeons who may be concerned about proceeding with TKA in a patient with valgus deformity^15^.

Our study also found that poor preoperative range of motion was not a significant predictor for periprosthetic fractures following primary TKA. It has been described in previous studies that poor pre-operative range of motion correlated with poor post-operative ROM and poorer functional outcomes. However, these poorer outcomes appeared not be the caused by PPF, but probably from poorer mobility and chronic pain from stiffness^16^.

Surgery done on the left knee was found to be significant at p=0.085 on univariate logistic regression and still significant on a multivariate logistic regression (p=0.0169, odds ratio 2.88). The relevance of side of surgery is currently unknown and more work is needed to determine if this correlates with the side of leg and what the implications are for performing surgery on the non-dominant leg.

A previous biomechanical study looking at muscle strength in dominant and non-dominant legs of females found that there is significant asymmetry in leg muscle strength, with stronger flexion in the non-dominant leg but stronger extension in the dominant leg^17^. There is little in the way of published research that investigates the significance of the side of surgery and subsequent rates of fractures or complications.

Young *et al* in 2013^18^ who investigated the impact of leg dominance on balance recovery, found that when subjected to a force which caused them to fall or become off balance, patients did not seem to fall more frequently if pulled towards their non-preferred side. However, this hypothesis was tested on normal patients and not those who had undergone surgery. Our study showed an association with the side of surgery (left side) and periprosthetic fractures; however, we were unable to ascertain whether this correlated with leg dominance.

Ten (23.8%) of our patients with periprosthetic fractures had anterior femoral notching after primary TKA. This was found to be significantly higher than our control group (p=0.0019, odds ratio 10.66). Femoral notching has been found to significantly decrease distal femoral torsional load to failure in biomechanical studies^19-20^. However, some clinical studies looking at the effect of femoral notching have reported that there is no increase in the rate of occurrence of supracondylar fractures in the presence of notching^10^. Our study has shown anterior femoral notching to be a significant risk factor in periprosthetic fractures, in contradiction to the findings of Ritter *et al* in 200521.

A major difference between the previous studies and our study is that previous studies identified a cohort of patients who underwent total knee replacement and subsequently followed them up to look for periprosthetic fractures, whereas our study initially identified a cohort of patients with periprosthetic fractures. Unfortunately, this methodology resulted in a very small number of periprosthetic fractures in existing studies (two fractures out of 1089 patients for Ritter *et al*, and three fractures out of 200 patients for Gujarati *et al*)^21,10^ – which may make the applicability of their findings limited.

In contrast, our study looked at a much larger cohort of 42 patients with periprosthetic fractures. When compared to the non-fracture group, it is clear that anterior femoral notching was higher in the fracture group, with a periprosthetic fracture being 17 times more likely to occur in a patient with anterior femoral notching.

A subgroup analysis for all these pre-operative mechanical factors was also done – pre-op BMI, diagnosis of OA or RA, pre-op alignment and pre-op range of motion were not found to be significant predictors of late or early periprosthetic fractures. However, the side of surgery was significantly higher in the early PPF group (p=0.0407). This further confirmed that pre-operative factors like alignment, range of motion and BMI were not likely to have significant effects on periprosthetic fractures, but again highlighted this interesting finding of left sided TKAs being more likely to sustain fracture.

Our study has several strengths: our matched patient groups (controlled for age, gender and BMI) allowed us to minimize confounding factors. In addition, study data was prospectively collected via our hospital registry and this allowed minimisation of recall bias. To our knowledge, there are few studies in current literature which analyse the association between side of surgery, coronal alignment and the incidence of PPF.

The limitations of our study is that of a relatively small sample size (though power analysis revealed our study to be adequately powered). In addition, not all patients had their bone mineral density measured and a large majority of our patients were female; and therefore likely to also have underlying osteoporosis. This bias was minimised by matching the control group for age and gender.

Additionally, both groups of patients were not heterogeneous in terms of implant type (CR or PS implant), resurfacing of patella or surgical approach, which could represent possible confounders. Although the numbers of CR and PS implants were significantly different between the fracture and non-fracture groups, previous studies have shown no difference in rate of periprosthetic fractures for CR or PS implants^22^.

## Conclusion

Periprosthetic fractures are largely not influenced by preoperative mechanical factors like coronal alignment and range of motion, but rather by intra-operative technical events, specifically anterior femoral notching. Hence, careful emphasis on operative techniques could mitigate this devastating complication regardless of the pre-surgical mechanical alignment or range of motion.
